# Editorial: Rising stars in avian physiology: 2022

**DOI:** 10.3389/fphys.2022.1100835

**Published:** 2022-12-07

**Authors:** Massimiliano Petracci, Vincent M. Cassone

**Affiliations:** ^1^ Department of Agricultural and Food Sciences, Alma Mater Studiorum—University of Bologna, Bologna, Italy; ^2^ Department of Biology, University of Kentucky, Lexington, KY, United States

**Keywords:** avian physiology, rising stars, poultry science, ornithology, research, development-innovation

Recognizing the future leaders of Avian Physiology is fundamental to safeguarding tomorrow’s driving force in innovation. This Research Topic is aimed to supporting scientists in the early stages of their careers across a wide range of disciplines by selecting and publishing their research output at the highest quality standards. In some sense, Avian Physiology is an amalgam of several fields and disciplines. This is reflected in the fact that veterinary oversight of animal research often distinguishes birds from poultry, birds that are used in agriculture. On one hand, avian physiologists who study poultry with the important objective of improving the health and profitability of poultry species are frequently called “poultry scientists”. Poultry scientists have their own journals, such as Poultry Science, and their own societies, such as the Poultry Science Association and others. On the other hand, avian physiologists who study basic mechanisms in a variety of avian species, including poultry species, are often called “ornithologists”. Ornithologists, like poultry scientists, have their own journals, such as Auk and Condor, as well as societies such as the International Ornithological Congress. And there are societies, such as the International Society for Avian Endocrinology, and journals, such as this journal, that address scientific issues from the breadth of Avian Physiology. This Research Topic reflects that breadth.

The progressive expansion in poultry production over the last several decades to meet growing demand for food has attracted much concern due to the adverse effects of the most challenging environmental stressor, heat stress on birds. For broiler chickens, their physiological response to thermal challenge varies according on several factors; among them, strains’ genotype plays a key role. This is particularly true as the climate changes, especially in tropical and subtropical areas. In a couple of review articles, Brugaletta et al. and Teyssier et al. synthesize existing knowledge on the influence of heat stress on physiology, gut health, and live performance of chickens, with a special emphasis on nutritional strategies to be adopted for broiler chickens to mitigate the adverse effects of increasing temperature. Brugaletta et al. provide introductory knowledge on heat stress physiology to make good use of the nutritional themes covered by Teyssier et al. On the same topic, Malila et al. investigated the consequences of cyclic thermal stress in chickens differing in growth rate and muscle development from different breeds on histological traits, gene expression related to adipose infiltration and inflammation in pectoral muscles. It was clearly confirmed that cyclic thermal challenge negatively affects live performances and breast yields, but infiltration of adipose tissue and inflammatory cells were reduced when fast-growing chickens were considered.

In the last few decades, growing demand for poultry meat has favoured the expansion of the use of fast-growing and high-breast-yield hybrids. This has enormously increased the pressure on pectoral muscular growth rate and mass in meat-type chickens and turkeys, indirectly increasing the occurrence of growth-related muscle abnormalities, such as white striping, an infiltration of fat into muscle, wooden breast, a condition where breast muscle is hard to the touch, and spaghetti meat, a condition where breast muscle is soft and has lost integrity. Tasoniero et al. investigated the onset of protein degradation processes occurring during post-mortem time and evolution of water distribution in pectoral muscles affected by spaghetti meat during refrigerated storage. The softer consistency and looseness of muscle integrity in breast fillets affected by spaghetti meat are not associated with greater proteolysis in live muscle and during post-mortem aging. On the other hand, spaghetti meat condition negatively affects water holding capacity due to abnormal water distribution caused by fibre myodegeneration. On the same topic, Soglia et al. tested the expression and distribution of muscle-specific proteins (vimentin and desmin) over the growing period in fast- and medium growing chickens to explore the existence of a relationship between the occurrence of muscle regeneration associated with the occurrence of growth-related muscle abnormalities and the growth profile of chicken genotype. The higher expression level of the desmin gene in fast-growing chickens supported its potential application as markers of the regenerative processes occurring in pectoralis major muscle affected by these muscle abnormalities. By using a risk analysis approach, Bordignon et al. investigated the role of production factors on the occurrence of the above-mentioned growth-related muscle abnormalities by using a large dataset collected in different trials conducted under varying experimental conditions. It was found that breast yield is a potential risk factor for white striping, while an elevated growth rate plays a major role in the induction of wooden breast and spaghetti meat which exhibited different probability levels when gender was considered.

During the last decade, there is a growing interest in the utilization of insects as a source of proteins in poultry diets. Biasiato et al. investigated the provision of insect larvae from Black soldier fly and yellow mealworm as potential environmental enrichment strategy to promote welfare in broiler chickens. It was evidenced that provision of insect larvae stimulated foraging behaviour, activity levels was increased and some overall behaviours potentially attributable to frustration were reduced, while plumage status, leg health and excreta corticosterone metabolites were not affected.

On the ornithological side, Hope et al. addressed a classical biological debate about the question of nature (genetics) *versus* nurture (environment). In birds, one key issue of the early developmental environment is the management of conditions (e.g., temperature, relative humidity, gas composition, lighting, *etc.*) during embryo development and the post-hatching period. In their study, Hope et al. focuses their attention on genetic background and post-hatching environmental conditions using zebra finches (*Taeniopygia guttata*) as their model. The results clearly showed that metabolic rate during embryo development and post-hatch is affected by parental inheritance, but pre- and post-hatch environmental conditions are of utmost importance. The latter can become particularly relevant considering climate change. How thermoregulatory ability is affected by early thermal environment and parental care will likely be shaped by natural selection as temperature continues to climb.

The melanocortin receptors (MCRs) are recognized to be gene family in the rhodopsin class of G protein-coupled receptors (MC1R-MC5R) which are only found in bony vertebrates. MCRs and their accessory proteins (MRAPs) are implicated in a large number of physiological processes such as pigmentation, lipolysis, adrenal steroidogenesis, and immunology. Indeed, Zhang et al. explored the relationship between MRAPs and sensitivity of cMC5R to natural chicken melanocortin peptides and studied how metabolism of liver is modulated in chickens. The main new finding is that ACTH likely plays a direct role in the regulation of glycolipid metabolism in the liver; this opens new research vistas to better explain the function of MC5R in multiple avian species.

West et al. evaluated the use of advanced behavioral tests to be used for deepening the knowledge on the foraging tactile specialization in Pekin ducks, *Anas playrhynchos domestica,* and Muscovy ducks, *Carina moschata domestica,* which were selected for their different profiles. It was found the females of both species have better predisposition toward learning strategies adopted in behavioral tests compared with males. As expected, differences were found between Pekin and Muscovy ducks in tactile ability to discriminate between hard and soft objects.

Finally, Smiley et al. provide a perspective on the inherent barriers with current research methods that lead to these biases in avian endocrinology in which studies on male birds predominate studies of female birds and experimental designs. This is a historical bias that continues to this day. For example, most studies of avian courtship focus of the generation of male birdsong without regard to female song and reaction to male advances. Considering that the rate-limiting steps in avian reproduction is the energy necessary to lay eggs and incubate them, both field and laboratory ornithologists must address experimental designs that reflect female avian behavioural physiology and endocrinology.

